# Obituary for Efstratios (Stratis) Avrameas

**DOI:** 10.31138/mjr.33.4.480

**Published:** 2022-12-31

**Authors:** Haralampos M. Moutsopoulos

**Affiliations:** Academy of Athens

## In memoriam

On December 8, 2022, Efstratios (Stratis) Avrameas, a novel immunology researcher passed away in Paris. Stratis was born in Athens, on January 1^st^, 1930. He studied Chemistry at the National and Kapodistrian University of Athens, from where he graduated in 1954. He worked for two years at the Biochemistry Laboratory of the Hellenic Pasteur Institute, under the supervision of Zoi Mela-Ioannidis.

In 1960, with a scholarship from the French government, he was accepted in the Microbial Chemistry Laboratory under the direction of Professor Pierre Grabar, at the Institute Pasteur, in Paris. Four years later (1964), he was awarded the title of Doctor of Biological Sciences (Doctorat d’État), by Pierre and Marie Curie University, in Paris. In 1963, he was appointed Research Assistant by the National Center for Scientific Research (CNRS) of France, and worked in the Department of Protein Chemistry of the “Institute for Scientific Research on Cancer” in VilleJuif, at first under the direction of Pierre Grabar and then under the supervision of André Lwoff, a Nobel prize laureate. In this laboratory, he rose up the hierarchy and became Research Director. In 1972, he was invited by Jacques Monod, another Nobel laureate, to return to the Institute Pasteur, in Paris where he headed the Immunocytochemistry Unit until his retirement, in 1999. In 1976, he was named Professor at the Pasteur Institute in Paris. From 1999 to 2002, he served in the position of Scientific Director at the Biotechnological Company DIATOS, founded by the French Pasteur Institute. His efforts aimed at developing proteins and peptides capable of transporting substances into cells with biological actions. After retiring from the Director’s position at the Pasteur Institute in Paris, he returned to his homeland and worked voluntarily as a consultant both in the Immunology Laboratory of the Hellenic Pasteur Institute and the Immunology Laboratory of the Pathophysiology at the Athens University Medical School.

Professor Avrameas was a pioneer researcher of Immunology. He has published over 300 papers. At first, his research led to the coupling of an antibody to the enzyme peroxidase with the chemical agent glutaraldehyde. This achievement then, paved the way for the development of the enzyme-linked immunosorbent assay (ELISA).^1^ This method replaced the radio-immunoassay (RIA) and thus, eliminated the exposure to radioactivity of employees working in research and diagnostic laboratories. Stratis, using the ELISA method, examined sera from healthy volunteers and demonstrated that these sera contained multitude of antibodies which recognised antigens of the human body (self-antigens autoantigens) and non-self antigens, namely, the gut microbiota. These antibodies had multireactivity. He called them **natural autoantibodies**.^2,3^ Based on these findings, he coined the theory of **natural autoimmunity**, in other words, the ability of the immune system to “self-awareness” («γνώθι σ’&alpha;υτόν») before becoming able to recognise and fight the foreign invaders.^4,5^ Under physiological conditions, the auto-polyreactive natural autoantibodies have their active site blocked by the high amount of available self-antigens. In disease states, where self-antigens are released, the natural autoantibody network is a guardian that rapidly sequesters the released, from disease tissues, self-antigens. Moreover, these autoantibodies have vital homeostatic biological activities such as cellular repairing and enzymatic catalysis. Finally, based on our own experiments and Stratis’ ones, we formulated the hypothesis that autoimmune diseases are the result of the hyper-function of natural autoimmunity against a specific cell or organ.^6^

Stratis has been awarded the following significant prizes for his novel work:

- Prize of the Foundation of Dr K. and Mme Peyre of the French Academy of Sciences (1974),
- Celine Prize (1979),
- Knight of the French Order of Merit (1980),
- Gold Medal of the Indian Federation of Clinical Biochemistry (1981),
- State prize (Prix d’État) of the French Academy of Sciences (1985),
- Corresponding Member of the Academy of Athens (1985),
- Gold Award of the French Federation for the Promotion of Progress (1990),
- Officer of the French National Order of Merit (1990),
- Honorary member of the Hellenic Immunological Society (1991),
- Honorary member of the National Medical Academy of Mexico (1995),
- Medal of Honour of the French National Centre for Scientific Research (1997),
- Honorary Research Director of the French National Centre for Scientific Research (CNRS) (1997),
- Honorary Professor of the Pasteur Institute of Paris (1998),
- Honorary Doctorate Degree of Athens University Medical School (2003),
- Gold Cross of the Order of Honour from the President of the Hellenic Republic (2004).

We, who had the honour and pleasure to work with Stratis, will all remember his honest and powerful personality, his desire to promote scientific knowledge through research and his passion for helping younger colleagues to acquire scientific knowledge.

May his memory be eternal.

**Haralampos M. Moutsopoulos**

Professor Emeritus and Member, Academy of Athens
